# The infection risk scan (IRIS): standardization and transparency in infection control and antimicrobial use

**DOI:** 10.1186/s13756-018-0319-z

**Published:** 2018-03-09

**Authors:** Ina Willemsen, Jan Kluytmans

**Affiliations:** 1 Laboratory for Microbiology and Infection Control, Amphia Hospital, PO Box 90158, 4800 RK Breda, The Netherlands; 2 Center for Infectious Disease Expertise and Research (CIDER), Tilburg, The Netherlands; 3 Julius Center for Health Sciences and Primary Care, UMC Utrecht, Utrecht, the Netherlands

**Keywords:** Infection prevention, Antimicrobial resistance, Guidelines, Benchmarking

## Abstract

**Background:**

Infection control needs user-friendly standardized instruments to measure the compliance to guidelines and to implement targeted improvement actions. This abstract describes a tool to measure the quality of infection control and antimicrobial use, the Infection Risk Scan (IRIS). It has been applied in a hospital, several nursing homes and a rehabilitation clinic in the Netherlands.

**Method:**

The IRIS consists of a set of objective reproducible measurements, combining patient- and healthcare related variables, such as: hand hygiene compliance, environmental contamination using ATP measurements, prevalence of resistant microorganisms by active screening, availability of infection control preconditions, personal hygiene of healthcare workers, appropriate use of indwelling medical devices and appropriate use of antimicrobials. Results are visualized in a spider plot using traffic light colors to facilitate the interpretation.

**Results:**

The IRIS provided ward specific results within the hospital that were the basis for targeted improvement programs resulting in measurable improvements. Hand hygiene compliance increased from 43% to 66% (more than 1000 observations per IRIS, *p* < 0.000) and ATP levels were significantly reduced (p < 0.000). In the nursing homes, large differences were observed with environmental contamination as common denominator. Most remarkable were the difference in Extended Spectrum Beta-Lactamase Enterobacteriaceae (ESBL-E) prevalence (mean 11%, range 0–21%).

**Conclusion:**

The bundle approach and visualization of the IRIS makes it a useful infection prevention tool providing standardization and transparency. Targeted interventions can be started based on the results of the improvement plot and repeated IRIS can show the effect of interventions. In that way, a quality control cycle with continuous improvement can be achieved.

## Background

Healthcare-associated infections (HAI) constitute a major public health problem worldwide [1]. In the future, an increase in HAI is expected due to the growing number of vulnerable and elderly patients and the emergence in antimicrobial resistance [2, 3]. Therefore, it is of utmost importance to intensify our effort in infection control and antimicrobial stewardship [4]. There is no standard method to measure the quality of care on these aspects. Therefore, we developed the infection Risk Scan (IRIS). This is a standardized method that assesses the quality of infection control by measuring different patient (residents in nursing home)-, department- and care related risk factors. This bundle of measurements provides a complete picture, which is visualized in an easy to understand way to give healthcare providers insight in the strengths and weakness of their performance.

We describe the IRIS-method, it’s implementation and the results obtained in a hospital, several nursing homes and a rehabilitation clinic.

## Method

**Fig. 1:**
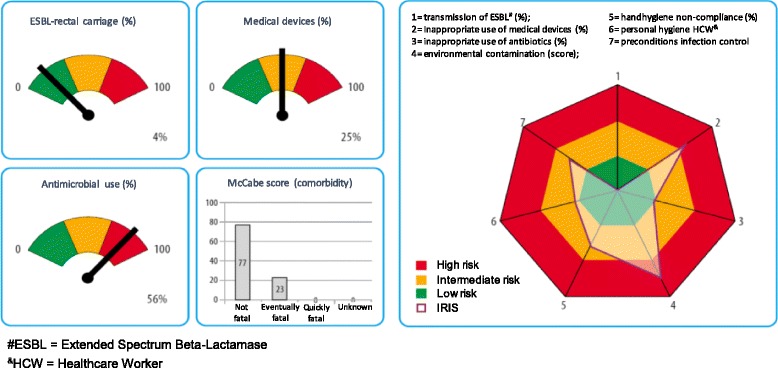
Example of the IRIS for hospitals. The left part of the figure shows the risk-profile, and the right part of the figure shows the improvement-plot

IRIS consists of cross-sectional measurements and investigates patient/resident-, ward- and care-related variables. Patient/resident-related risks were visualized in a patient risk profile. Ward and care-related risk were visualized in an improvement spider-plot (Fig. 1). Table 1 shows an overview of the included variables, methods, outcome measures and risk stratification.

**Table 1:** Overview of all collected variables, the method used, outcome variables that are visualized in the risk profile and improvement plot, and breakpoints for the risk classification

					Risk classification		
variables	Hospital	NH^^^	method	Outcome variable	Low	Intermediate	High
Risk profile							
Severity of underlying diseases, according to the McCabe score	X		Prevalence survey (file research & interview)	The percentage per category is presented in the risk profile	N.A.^&^		
Independency scale according to Katz-score		X	Prevalence survey (file research & interview)	The percentage per category is presented in the risk profile	N.A. ^&^		
indwelling medical devices, including venflon not in use	X		Prevalence survey (file research & interview)	Percentage (n=total number inclusions)	≤ 15	>15 and ≤50	> 50
		X			≤ 5	>5 and ≤10	> 10
Antibiotic use	X		Prevalence survey (file research & interview)	Percentage (n=total number inclusions)	≤ 15	>15 and ≤50	> 50
		X			≤ 5	>5 and ≤10	> 10
Rectal carriage of ESBL-E^ǂ^ and CPE^ǂǂ^	X	X	Prevalence survey (culture of faeces or perianal swab)	Percentage (n=total number patients of whom faeces was cultured)	≤ 7	>7 and ≤11	> 11
Improvement plot							
Inappropriate use of medical devices	X	X	Prevalence survey (file research & interview)	Percentage (n=total number of patients with medical devices in use)	≤ 15	>15 and ≤25	>25
Inappropriate use of antibiotics	X	X	Prevalence survey (file research & interview)	Percentage (n=total number of patients administered one or more antibiotics)	≤ 15	>15 and ≤25	>25
Transmission of ESBL-E and CPE	X	X	Prevalence survey (molecular typing of ESBL-E and CPE)	Transmission = 2 or more identical ESBL-E and CPE with an epidemiological link	No transmission	No transmission	Transmission
Hand hygiene non-compliance	X		Direct observations of hand hygiene moments per unit	% non-compliance overall (n>200 moments)	≤ 40	>40 and ≤60	>60
Environmental contamination	X	X	ATP detection on pre-defined surfaces/objects per ward. Per tested surface/object, RLU is converted to a score (1, 2, 3 or 4)	Total score per ward/setting (in case of multiple wards within 1 setting, breakpoints will be adjusted as needed)	≤ 4 ≤ 3	>4 and ≤12 >3 and ≤9	>12 ZH >9 VH
Shortcomings in infection control preconditions	X	X	10 preconditions are observed per ward	Score from 1 to 10	≤ 1	>1 and ≤3	>3
Personal hygiene of healthcare workers	X	X	20 nurses and other healthcare employees, per ward, were checked for compliance with the dress code.	Score from 1 to 20 (in case of less observations, breakpoints will be adjusted as needed)	≤ 1	>1 and ≤4	>4

^*^*^ *NH = Nursing Home;*

^&^N.A.=not applicable; ^ǂ^ ESBL-E = Extended Spectrum Beta-Lactamase producing Enterobacteriaceae

^ǂǂ^CPE-E = Carbapenemase producing Enterobacteriaceae

One or more point-prevalence surveys were performed by infection control practitioners according to the Dutch national surveillance protocol for hospitals (PREZIES) or nursing homes (SNIV) [5, 6]. All patient records of the included patients/residents were investigated, and if necessary, discussed with the attending physician. At least 50 patients (residents in nursing homes) were included in each IRIS.

### Risk profile

The risk profile shows the vulnerability of the patient population and consist of four variables:

1. Prevalence of indwelling urethral or suprapubic catheters and intravascular devices (including a peripheral intravenous catheter not in active use) on the day of the survey. The risk classification was based on prevalence numbers from the national surveillance for hospitals as well as for nursing homes [5–8].
2. Prevalence of intravenous or oral antibacterial antimicrobial therapy on the day of the survey [9]. Inhalation medication, cement beads, topical antibiotics, antiviral and antifungal therapy, were not included, nor did we include antibiotic prophylaxis administrated in the operating theater. The risk classification was based on prevalence obtained from the national surveillance for hospitals as well as for nursing homes [5, 6, 8, 9].
3. Prevalence of rectal carriage of Extended Spectrum Beta-Lactamase (ESBL)-producing Enterobacteriaceae (ESBL-E), detected through perianal swab culture (Table 1) [10, 11]. Rectal ESBL-carriage is common in the population. In Dutch nursing homes a range in ESBL-E prevalence between zero and 21% was found [8, 12]. The average prevalence in the Amphia hospital was 5% in the last 4 years, with a high variability in genotypes [13]. Considering the inaccuracy of the measurement, a prevalence of 7% or lower was interpreted as low.
4. Expected mortality and comorbidity, expressed in a graph showing the distribution in McCabe scores in hospitals, or dependency in activities of daily living in nursing homes according to the Katz Index [14, 15].

### Improvement plot

The improvement plot shows 7 ward- and care-related risk factors, both process- as well as outcome variables. These factors can be influenced by the healthcare professional or organization.

1. Appropriate use of indwelling medical devices

Appropriateness of the indication for intravascular devices was judged based on local guidelines. The appropriateness of the indication of urethral catheters was judged according to the flowchart used in the national Dutch prevalence survey for HAI [5]. The proportion of patients with a medical device that was considered inappropriate was presented in the improvement plot. The cutoff points for classification are based on “expert opinion” as there are no reference values available.

2. Appropriate use of antimicrobial therapy

Appropriateness of treatment (indication and choice of antimicrobial) was judged against the local antibiotic formulary using a standardized method [9]. The following classifications were used: appropriate use (i.e. justified use and appropriate choice), inappropriate use (i.e. unjustified use and/or justified use, but inappropriate choice), or insufficient information. The proportion of patients with antimicrobial therapy that was considered unjustified and/or inappropriate choice was presented in the improvement plot.

3. Clonal relatedness of ESBL-E

Clonal relatedness was determined based on the microbiological cultures, ESBL gene detection using the Check-MDR CT103 microarray (Check-Points, Wageningen, Netherlands), and molecular typing using Amplified Fragment Length Polymorphism (AFLP) and epidemiological investigation [11, 16]. When two or more identical ESBL-E strains, with identical resistance genes were detected in two or more patients from one prevalence survey within the same epidemiological setting, this was judged as indicative for transmission. We assumed that one case per cluster was the index.

4. Environmental contamination

Detection of Adenosine Triphosphate (ATP) was used to identify the level of environmental contamination with organic material [17, 18]. Samples were taken, using an ATP device (3 M Inc., St. Paul, MN, US), from a fixed amount of pre-defined objects or surfaces, within each unit, at least two hours after the routine cleaning and in accordance to the manufacturer’s guidelines (Table 2). In hospitals and in nursing homes, 20 and 15 items were tested, respectively. These test points were selected because they met the following criteria: frequently touched objects/instruments by the nursing staff, frequently touched objects/surfaces by the patient; the immediate surroundings of the patients or items that should always be clean. For each test point, the amount of RLU was converted to a score:

**Table 2:** Overview of all tested surfaces and objects for environmental contamination in the hospital and nursing home

Testpoints	Hospital	Nursing Home
Bedrail (twice, in two rooms)	X	X
Over bed table	X	X
Washstand	X	X
Shower chair	X	
Support bar in the toilet room	X	X
Toilet seat (sitting area)	X	X
Door handle nursing office	X	
Patient alarm bell	X	
I.V. pole (most frequently touched part of the pole)	X	
Keyboard P.C. in the nursing office	X	X
Telephone	X	X
Control panel bedpan washer	X	
Bedside commode	X	X
Cabinet for medical supply & bandages	X	X
Blood pressure cuff	X	
Ear thermometer (ear tip)	X	X
Glucometer	X	X
Work surface of the bench for drug preparation	X	
Keyboard computer on wheels (COW)	X	
Table living room		X
Supply room “sterile” materials		X
Door handle living room		X
Patient lift, client handle		X

< 1500 RLU = 0 points; > 1500 and ≤3000 RLU = 1 point; > 3000 and ≤10.000 RLU = 2 points; and > 10.000 RLU = 3 points. The total score of all measured objects tested within the unit was presented in the risk plot. If more than one ward was monitored, results were adjusted proportionally (Table 1). The classification is based on an analysis of previous measurements.

5. Shortcomings in infection prevention preconditions

Several preconditions are essential for an effective infection control policy. The tested items are listed in Table 3, scoring and breakpoints are shown in Table 1.

6. Personal hygiene of healthcare workers

**Table 3:** Overview of all tested infection control preconditions in the hospital and nursing home

Infection control preconditions	Hospital	Nursing Home
Trash bin(s) are closed and foot-operated (entire department)	X	X
The (clean) linen is stored in a clean place, protected against dust and moisture	X	X
The bed-pan washer meets the following requirement: Disinfection with steam or hot water of at least 80 ° C (for at least 60 s)	X	X
Sterile medical devices (catheters, IVs) are kept in a closed cabinet	X	
Sterile medical devices are kept separated in a closed cabinet		X
Medical supply and bandages are kept in closed cabinets	X	
Needle waste container (UN3291) is presence		X
Halter aprons to protect clothing are present at the ward	X	X
Surgical masks are present at the division	X	X
Non-sterile gloves (NEN-EN 420 + A1, NEN-EN374, NEN-EN) are present in every patient room	X	
Non-sterile gloves (NEN-EN 420 + A1, NEN-EN374, NEN-EN) are present in every ward		X
Hand alcohol (is present in every patient room and at point of care (EN1500)	X	
No fabric chairs or benches are present in the patient and / or treatment room	X	
No fabric chairs or benches are present in the common areas		X

At least 20 healthcare workers (10 nurses, 5 staff physicians or house officers and 5 other hospital employees in hospitals; in NHs at least 20 healthcare workers overall) were tested for the basic hygiene rules: no rings, no watch or wrist jewelry present, forearms uncovered (bare below the elbow), uniform worn correctly and coat closed (Table 1).

7. Hand hygiene compliance

In the hospital, the hand hygiene compliance was determined by performing direct observation on the ward according to the World Health Organization 5 moments method. The observations were performed by trained nurses during routine handlings. The classification is based on scientific publications [19–21].

8. Presence of local infection prevention protocols

In the nursing homes, the presence of 20 essential infection prevention protocols was investigated (Table 4). These protocols contain essential basic principles of infection prevention. The rating is assigned based on “expert opinion” where one deviation is accepted.

**Table 4:** Overview of the infection prevention protocols, tested for local presence

Infection control protocols
Accidental blood contact
Collection and transport of waste and used linen
Hand hygiene
Pets (including assistance dogs)
Infectious diseases healthcare workers
Catheterization
Legionella management plan
Body care of the client
Recommendation for prevention & control of influenza
Recommendation for prevention & control of norovirus
Recommendation for prevention & control of Scabies
Recommendation for screening of Multi Drug Resistant Organisms (including screening of risk-population on admission^#^)
Recommendation for prevention & control of MRSA (including screening of risk-populations on admission^#^)
Storage of “sterile” materials
Personal Protection Materials (PPM)
Personal hygiene of healthcare workers
Cleaning, disinfection and sterilization
Administering medication
Urination and bowel movement (defecation)
Wound-care

# risk population as defined by the Dutch Working Infection Control Party

The selection of risk factors was based on the importance, as judged by a group of experienced infection control practitioners, as well as the possibility of an objective and reproducible assessment. The current set of risk factors are considered to be important for current infection control, however the IRIS is a flexible model in which risk factors can be added or switched.

For each risk factor, breakpoints were set to distinguish low, intermediate and high categories (Table 1). Breakpoints were based on scientific publications or based on expert opinion if no such data were available, shown in Table 1.

To visualize all surveillance data in one graph, data were converted into a scale from 0 to 100 using an algorithm. The algorithm included breakpoints for the 3 categories: low risk from 0 to 33%; intermediate risk from 34 to 66%; and high risk from 67 to 100%. Each axis of the plot represents an outcome variable or risk factor. If the results were in the high (red) risk-area, in-depth research and improvement activities were strongly recommended.

The risk profile and improvement plot were reported to the management of the hospital ward or healthcare setting. Management itself is responsible for the distribution of the results to all employees and for the implementation of improvement actions. The infection control department had a coaching and consulting role during the improvement programs. The figure of the improvement plot, in combination with the patient profile, gives direction to the improvement activities and helps to set priorities. The risk profile provides background information about the population. This is helpful for the interpretation of the improvement-plot and subsequent improvement activities. In a high risk population, with high prevalence of medical devices and severity of underlying diseases, environmental contamination and low compliance of hand hygiene is more critical than in a low risk population.

## Results

### IRIS in a hospital

**Fig. 2:**
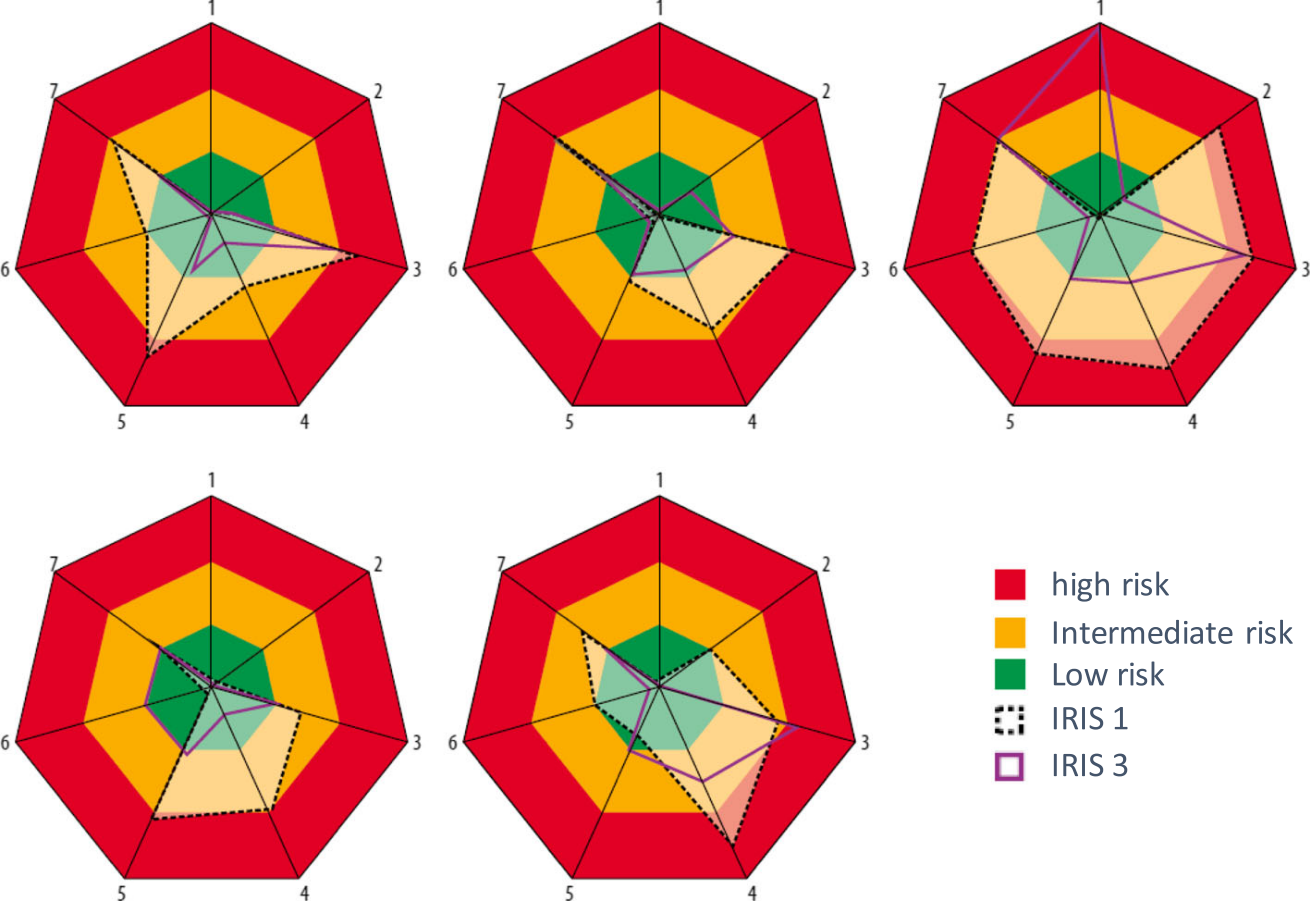
The Improvement-plots from 5 hospital units, from 5 different medical specialties. Three IRIS-cycle were performed with an interval of 6 up to 8 months. The dotted black line shows the results from the first IRIS, the purple line shows the results from the third IRIS. 1= Transmission of ESBL (%); 2= Inappropriate use of medical devices (%); 3= Inappropriate use of antibiotics (%); 4= Environmental contamination (score); 5= Hand hygiene non-compliance (%); 6= Personal hygiene Healthcare workers (%); 7= Preconditions infection control (score)

IRIS was implemented in 5 wards within 5 different medical specialties during the period 2013–2015. Three cycles of measurements, improvements and measurements were performed during this period with an interval of 6–8 months. Differences were found in the figures of the improvement plots of the different wards. However, high levels of environmental contamination were found in all 5 wards (Fig.2).

Based on the results, targeted actions were performed resulting in a considerable improvement (hand hygiene dispensers at point of care, hand hygiene campaign for and by nurses, definition of cleaning responsibilities). Especially, hand hygiene compliance improved from an average of 43% to 66% (*p* < 0.001), and the measured ATP levels reduced significantly (p < 0.001) meaning a cleaner environment. Of all tested patients (*n* = 439), 16 (3.6%) were proven to be carrier of a ESBL-E in the perianal culture. In two patients, an identical strain was found. No carbapenemase-producing Enterobacteriaceae were isolated. No improvement was detected in the use of antimicrobials.

### IRIS in a nursing home

IRIS was performed in 19 nursing homes in the southern part of The Netherlands. Large differences were found between the nursing homes and again the common denominator was the environmental contamination. Furthermore, in most of the nursing homes, availability of hand disinfectants was insufficient and the separation between clean and dirty material was lacking. The most remarkable finding was the difference in ESBL-E prevalence, mean 11% (range 0–21%). A large outbreak was detected in one nursing home, involving more than 21 residents in different departments, with an identical *Escherichia coli* ST131 strain. [12]

### IRIS in a rehabilitation center

In this center, 12 out of 71 (17%) cultured residents proved to be a carrier of ESBL-E. The molecular typing revealed that all ESBL-E were unique cases, no clonal clusters could be detected. In this center, all variables in the IRIS improvement plot were in the “green” zone. Environmental contamination was minimal, infection prevention preconditions were good and local protocols were available.

### Implementation of the IRIS

The time needed to perform an IRIS depends on the size, setting and condition of (electronic) patient records. To complete an IRIS in a hospital ward with 50 beds and electronic patient records takes approximately 5 days, of which 3 days are for the measurements and 2 days for analyses and feedback. An IRIS in a nursing home with 100 residents takes about one day for preparation and execution of the prevalence survey, one day for audits, and one day for analysis and feedback. This does not include the time investment for the hand hygiene observations. Our experience reveals that during the morning routine, on average, 20 hand hygiene moments take place per hour.

Management and healthcare workers were very positive about the IRIS process. The healthcare workers on each ward implemented changes based on the IRIS results and the collaboration between the infection control department and the ward staff was considered constructive. In the hospital, infection prevention workgroups were developed to improve shortcomings together. This resulted in significant improvement in all departments.

## Discussion

We describe the implementation and first results of the IRIS, an infection prevention tool that uses a bundle approach and provides transparency in the performance of infection control and use of antimicrobial therapy. Measurements are objective, reproducible, and include the most relevant indicators of infection control and antimicrobial resistance. The IRIS has been performed in 19 nursing homes, 1 rehabilitation center and 5 wards within one hospital.

The difference in improvement plots revealed that each hospital ward or healthcare setting has specific issues that need improvement. Targeted interventions were undertaken based on the IRIS results. The second and third IRIS showed an overall improvement in the hospital, with the exception being the appropriateness of antimicrobial use. This can be explained by the fact that no stewardship activities were initiated except for feedback of the results. A more intensive approach will be needed to obtain measurable improvement.

In the nursing homes, significant variation in the IRIS components was found, most remarkable were the differences found in ESBL prevalence. In one setting, a large outbreak was detected. The use of diagnostic tests, such as microbiological cultures, is limited in most nursing homes. This could result in a possible reservoir for multidrug-resistant microorganisms. Prevalence surveys, like the prevalence survey in the IRIS, are useful tools to detect the possible clusters of ESBL-E at an early stage.

The IRIS components are not new, and well known of infection prevention and antibiotic stewardship programs. However, bringing these various components together, like a bundle, results in stronger effect than those of the individual interventions. Furthermore, the multifactorial measurements are objective and reproducible, which makes comparison between healthcare settings possible.

The color codes make the results straightforward and easy to understand, for professionals who need to implement the improvements and for the managers who should promote and monitor the activities. The simplicity of the visualization creates co-ownership of the problem and provides support for interventions.

In the current IRIS, hand hygiene compliance was measured by performing direct observations of healthcare workers during the morning routine. Direct observations can lead to unrealistic high compliance, known as the Hawthorne effect. Furthermore, it gives information about a very small portion of the healthcare workers during a small time-frame of the day. We recognize these limitations, however, it is not easy to solve this issue. The use of consumption volumes of hand sanitizer could be an alternative; however, this first needs to be validated.

In the IRIS, appropriateness of use of medical devices and antimicrobial therapy were judged based on local guidelines. Local guidelines may vary and this may limit the use of this method when comparisons are made between centers. Standardization of guidelines is needed to be able to use IRIS or similar methods on a larger scale.

Furthermore, thresholds were, where possible, based on data from surveillance programs or peer-reviewed publications, but when no references were available thresholds were chosen arbitrary by a group of experts. In these cases previous results were used to define thresholds. The threshold values should be evaluated periodically and adjusted if necessary.

## Conclusion

In conclusion, the bundle approach and visualization of the IRIS makes it a complete and useful infection prevention tool. It provides transparency in the quality of infection control and antimicrobial use. Targeted interventions can be started based on the results of the improvement plot and the effect of interventions can be shown by repeating the IRIS. In that way, a quality control cycle with continuous improvement can be achieved. The broader implementation of IRIS can raise the standard of infection control and make it more transparent in various healthcare settings, E.g. Nursing homes.

## Abbreviations

AFLP: Amplified Fragment Length Polymorphism
ATP: Adenosine Triphosphate
ESBL: Extended Spectrum Beta-Lactamase
ESBL: Extended Spectrum Beta-Lactamase
HAI: Healthcare Associated Infections
IRIS: Infection Risk Scan

## References

[1] Allegranzi B, Nejad S, Combescure C, Graafmans W, Attar H, Donaldson L, Pittet D. Burden of endemic health-care-associated infection in developing countries: systematic review and meta-analysis. Lancet. 2011;377:228–41. 10.1016/S0140-6736(10)61458-4 21146207

[2] European commision, Europe in figures, eurostat yearbook 2010. http://ec.europa.eu/eurostat/documents/3217494/5721265/KS-CD-10-220-EN.PDF/e47b231c-c411-4d4e-8cd6-e0257be4f2e6?version=1.0 Accessed 1^st^ Nov 2017.

[3] Strausbaugh LJ (2001). Emerging health care-associated infections in the geriatric population. Emerg Infect Dis.

[4] Moro ML, Gagliotti C (2013). Antimicrobial resistance and stewardship in long-term care settings. Future Microbiol.

[5] PREZIES (2016). Prevalentieonderzoek Ziekenhuizen: Protocol & dataspecificatie, versie maart/oktober 2016.

[6] SNIV Surveillance Netwerk Infectieziekten Verpleeghuizen: Protocol & dataspecificatie versie april/november 2016. Bilthoven: Rijksinstituut voor Volksgezondheid en Milieu; 2016. [in Dutch].

[7] Eilers R, Veldman-Ariesen MJ, Haenen A, Van Benthem BH. Prevalence and Determinants associated with healthcare-associated infections in long-term care facilities (HALT) in the Netherlands, may to June 2010. Euro Surveill. 2012;17(34):1-8. 22939212

[8] Willemsen I, Nelson-Melching J, Hendriks Y (2014). Measuring the quality of infection control in Dutch nursing homes using a standardized method; the infection prevention risk scan (IRIS). Antimicrob Resist Infect Control.

[9] Willemsen I, Groenhuijzen A, Bogaers D, Stuurman A, van Keulen P, Kluytmans J (2007). Appropriateness of antimicrobial therapy measured by repeated prevalence surveys. Antimicrob Agents Chemother.

[10] Kluytmans-van den Bergh MK, Verhulst C, Willemsen LE, Verkade E, Bonten MJ, Kluytmans JA (2015). Rectal carriage of extended-spectrum-beta- lactamase-producing enterobacteriaceae in hospitalized patients: selective preenrichtment increases yield of screening. J Clin Microbiol.

[11] Bernards AT, Bonten MJM, Cohen Stuart J (2012). NVMM guideline: laboratory detection of highly resistant microorganisms (HRMO).

[12] Willemsen I, Nelson J, Hendriks Y (2015). Extensive dissemination of extended spectrum Beta-lactamase producing Enterobacteriaceae in a Dutch nursing home. Infect Control Hosp Epidemiol.

[13] Willemsen I, Oome S, Verhulst C, Pettersson A, Verduin K, Kluytmans J (2015). Trends in extended Spectrum Beta-lactamase (ESBL) producing Enterobacteriaceae and ESBL genes in a Dutch teaching hospital, measured in 5 yearly point prevalence surveys (2010-2014). PLoS One.

[14] Reilly JS, Coignard B, Price L, Godwin J, Cairns S, Hopkins S, et al. The reliability of the McCabe score as a marker of co-morbidity in healthcare-associated infection point prevalence studies. J Infect Prev. 2015;20(8):127-29. 10.1177/1757177415617245 PMC5074213 28989468

[15] Katz S, Ford AB, Moskowitz RW, Jacksons BE, Jaffe MW (1963). Studies of illness in the aged: the index of ADL: a standardized measure of biological and psychosocial function. J Am Med Assoc.

[16] Mohammadi T, Reesink HW, Pietersz RN, Vandenbroucke-Grauls CM, Savelkoul PH (2005). Amplified-fragment length polymorphism analysis of Propionibacterium isolates implicated in contamination of blood products. Br J Haematol.

[17] Sherlock L, O’Connell N, Creamer E, Humphreys HP (2009). Is it really clean? An evaluation of the efficacy of four methods for determining hospital cleanliness. J Hosp Infect.

[18] Boyce JM, Havill NL, Dumigan DG, Golebiewski M, Balogun O, Rizvani R (2009). Monitoring the effectiveness of hospital cleaning practices by use of an adenosine triphosphate bioluminescence assay. Infect Control Hosp Epidemiol.

[19] Pittet D, Hugonnet S, Harbarth S, Mourouga P, Sauvan V, Touveneau S (2000). Effectiveness of a hospital-wide programme to improve compliance with hand hygiene. Infection Control Programme Lancet.

[20] Cooper BS, Medley GF, Scott GM (1999). Preliminary analysis of the transmission dynamics of nosocomial infections: stochastic and management effects. J Hosp Infect.

[21] McBryde ES, Pettitt AN, McElwain DL (2007). A stochastic mathematical model of methicillin resistant Staphylococcus Aureus transmission in an intensive care unit: predicting the impact of interventions. J Theor Biol.

